# Educational inequalities in disability among older adults: a trend analysis in 15 European countries (2002 to 2023)

**DOI:** 10.1038/s41598-026-62524-0

**Published:** 2026-07-20

**Authors:** Olaf von dem Knesebeck, Daniel Lüdecke

**Affiliations:** Institute of Medical Sociology, University Medical Center Hamburg Eppendorf, Martinistr. 52, 20246 Hamburg, Germany

**Keywords:** Education, Disability, Time trend, Europe, Gender, Inequalities, Health care, Health humanities

## Abstract

The aim of this study was to examine the magnitude and trends of educational inequalities in disability among older men and women (≥ 60 years) in 15 European countries. We used data of 11 waves of the European Social Survey (ESS, 2002 to 2023, N = 98,300). Disability was assessed by a variant of the Global Activity Limitation Indicator. In the latest wave of the ESS (2023), there was an educational gradient in the prevalence of disability in the 15 European countries. In terms of time trends among men, disability significantly declined in high education groups in ten countries while it increased among those with low education in three countries. Trends in prevalence were quite different among women. There was a significant decline among low as well as high education groups in five countries and prevalences were stable in eight countries. Overall, among men, there was a significant increase of inequalities in disability between low and high educational groups from 2002 to 2023, while this trend was not significant among women. The mechanisms behind these inequalities in disability are not yet fully understood. Future studies should investigate to what extent behavioural, psychosocial, and material factors contribute to their explanation. A better understanding of these explanatory factors is needed to develop and implement interventions for a reduction of inequalities in disability in aging societies.

**Supplementary Information:**

The online version contains supplementary material available at 10.1038/s41598-026-62524-0.

## Introduction

There is considerable evidence that education is an important social determinant of population health^1–4^. Accordingly, people with a lower education experience worse health outcomes compared to those with a higher education. Such educational inequalities in health have been found to persist among older age groups in European countries^5^. As the older population is growing rapidly in Europe, research on educational inequalities in health among the aged is needed as it can help to find strategies to promote health equity and healthy aging.

Disability and functioning are frequently used health indicators, especially in studies among the growing population of older adults^6,7^. The International Classification of Functioning, Disability and Health^8^ provides a conceptual basis for the definition and measurement of functioning and disability^9^. According to this framework, disability is an umbrella term for impairments, activity limitations and participation restrictions that often result from chronic conditions. While impairments reflect problems in body function and structure, activity limitations indicate difficulties in executing activities, and participation restrictions mark problems in the performance of roles and social involvement. Given their relevance, studies that help to understand inequalities and trends in disability are crucial. In terms of the latter, a recent analysis of time trends in activity limitations across 30 European countries showed that the prevalence of limitations generally tended to decrease in older adults (≥ 65 years) between 2002 and 2018^10^. However, other studies found diverging trends across countries^11–13^.

International studies suggest that there are educational inequalities in disability in older populations. For example, Fonseca et al.^2^ found that an additional year of education is significantly associated with experiences of limitations using data from 14 OECD countries among people aged 50 years and over. Based on seven waves of the Survey of Health, Ageing, and Retirement in Europe (SHARE), Almomani and Al-Masaeid^14^ recently analysed the relationship between education and difficulties in instrumental activities of daily living (IADL) in seven European countries. Results indicated that higher education is significantly associated with reduced IADL difficulties. However, country and gender differences emerged in the relationship. Although the mechanisms behind these inequalities in functional limitations have hardly been examined, it is known that most chronic diseases are more prevalent among the lower education groups^15^ and that limitations often result from chronic conditions. It is likely that education as a resource provides cognitive abilities and personality profiles which shape health-related behaviour and influence the accessibility to better environmental conditions, thereby reducing the risk of both, chronic diseases and disability^16^. In terms of country differences in educational inequalities, it can be expected that structural and cultural factors like welfare states, educational systems, cultural norms, and health behaviours play a role. These factors create diverse societal environments where educational inequalities translate differently into chronic diseases and disability^13^.

There are a few studies analysing trends of educational inequalities in disability in Europe. One study examined inequalities in disability in 26 European countries between 2002 and 2017 among individuals aged 30 to 79 years^17^. The authors found that inequalities tended to increase over time. However, there was heterogeneity in these trends between the regions in Europe. Wilkie et al.^13^ analysed the trend of educational inequalities in functional limitations across 11 high-income European countries (plus, Northern America, China, and Mexico) at two time points (2004 and 2018). They found a significant increase of inequalities among people aged 65 years and older in three European countries (Austria, England, and Germany) and a significant decrease in Spain, while there were no significant changes in the remaining seven European countries. The authors pointed out that the widening inequalities had varying reasons: They were due to increased limitations among the least-educated and/or due to declines in limitations among the most-educated^13^. Overall, further research on trends in educational inequalities in disability among older populations in Europe is needed as this is an important aspect of healthy aging. In this regard, analyses of recent trends are missing. Moreover, there is not much known about gender differences. Against this background the following research questions will be addressed: (1) What is the magnitude of educational inequalities in disability among older adults in 15 European countries according to recent available data? (2) How did the prevalence of disability in different educational groups develop between 2002 and 2023? (3) Did the educational inequalities in disability change between 2002 and 2023? (4) Are there country and gender differences regarding these trends?

## Methods

### Data

We used data of the 11 waves of the European Social Survey (ESS; 2002, 2004, 2006, 2008, 2010, 2012, 2014, 2016, 2018, 2020, and 2023). The ESS is a repeated cross-sectional investigation conducted in a varying number of European countries. Since 2002, every two years, representative samples of people aged 15 years and older in the participating countries are drawn. Individuals are selected by strict random probability methods at every stage. Data are predominantly based on face-to-face interviews with standardised questionnaires. Data and a comprehensive methodological documentation are available online^18^. The ESS subscribes to the Declaration on Ethics of the International Statistical Institute^18,19^. Accordingly, participants are protected against potentially harmful effects of participating in research and participation in the ESS was based on a freely given informed consent.

In this study, 15 European countries were included from which data of all 11 waves were available (i.e., Belgium, Switzerland, Germany, Spain, Finland, France, Great Britain, Hungary, Ireland, the Netherlands, Norway, Poland, Portugal, Sweden, and Slovenia). For the present analyses, a subsample of all people aged 60 years or older in the 15 countries was used (total in the 11 waves: N = 98,300). This subpopulation was chosen because we are particularly interested in educational health inequalities in older populations. Moreover, the prevalence of disability is quite low in the younger age groups. We decided to use the 60 years cut-off as this is the definition of old age used by the United Nations^20^.

### Measures

Educational attainment was measured by using country-specific questions and was then recoded into the International Standard Classification of Education (ISCED) scale^21^ according to guidelines developed for the ESS^22^. ISCED provides a globally agreed framework for categorising education programmes and qualifications and has been implemented in many EU data collections. While ISCED focuses on the type of qualification obtained and its position in the educational hierarchy, the alternative measure ‘years of education’ adds up the nominal duration of the programs attended or grades completed. For the analyses, the ISCED scale was recoded into three categories: low (primary and lower secondary education, ISCED 0–2), medium (upper secondary and post-secondary non-tertiary education, ISCED 3–4), and high (tertiary education, ISCED 5 and higher). In the 11 waves of the ESS, disability was assessed by the question: ‘Are you hampered in your daily activities in any way by any longstanding illness, or disability, infirmity or mental health problem?’, with the answer categories ‘yes, a lot’, ‘yes, to some extent’ and ‘no’. Similar questions are a frequently used indicator for population health monitoring in Europe, also referred to as the Global Activity Limitation Indicator (GALI)^17^. Like in previous studies, also using ESS data^16,17^, the categories ‘yes, a lot’ and ‘yes, to some extent’ were combined to indicate disability, whereas ‘no’ indicates no disability.

### Data analysis

Descriptive statistics for the 15 countries are presented using pooled data, weighted by respondent-level post-stratification weights, across all survey waves. Categorical variables are reported as proportions and numeric variables as means. The range across different survey waves is indicated by providing the lowest and highest observed values.

To estimate the prevalence of disability in the most recent ESS wave (2023), logistic regression models were fitted separately for each country. A global omnibus test was conducted to determine whether the prevalence of disability differed significantly across three educational levels.

To analyze time trends, two logistic multilevel regression models, one for women and one for men, were fitted. These models included an interaction term between education and survey wave. A country-indicator variable was used as a higher-level (level-2) random effect to account for country-specific variations in the outcome. Additionally, a random slope for the survey wave was included at the country level to capture variations over time across nations. These level-2 random effects were modelled using an unstructured variance–covariance matrix, with three variance parameters being estimated: the variance of the random intercept, the variance of the random slope for the survey wave, and the covariance (correlation) between the random intercepts and random slopes (Eqs. 1 and 2).

1 $$\begin{aligned} & \log it\left( {p_{ij} } \right) = \beta_{0} + \beta_{1} \left( {wave_{ij} } \right) + \beta_{2} \left( {education_{ij} } \right) \\ & \quad + \beta_{3} \left( {wave_{ij} \times education_{ij} } \right) + \left( {age_{ij} } \right) + u_{0j} + u_{1j} \left( {wave_{ij} } \right) \\ \end{aligned}$$

2 $$\begin{array}{*{20}c} {\left( {\begin{array}{*{20}c} {u_{0j} } \\ {u_{1j} } \\ \end{array} } \right)\sim \left( {\left( {\begin{array}{*{20}c} 0 \\ 0 \\ \end{array} } \right),\left( {\begin{array}{*{20}c} {\tau_{00}^{2} } & {\tau_{01} } \\ {\tau_{01} } & {\tau_{11}^{2} } \\ \end{array} } \right)} \right)} \\ \end{array}$$

Because we employed logistic regression models, the level-1 residual variance is theoretically fixed to $\pi^{2} /3$ and therefore not estimated from the data.

Based on these multilevel models, country-specific predicted probabilities were calculated (including 95% confidence intervals (CI)) to illustrate the estimated prevalence of disability across educational levels over time. To assess the statistical significance of the time trend slopes, average marginal effects were calculated. Furthermore, contrasts in average marginal effects between the lower and higher education groups were assessed. This analysis aimed to determine how the prevalence of disability changed between these two groups, thereby indicating whether educational inequalities increased or decreased over time. All regression models, including those for the most recent wave, were adjusted for age and weighted using post-stratified design weights. In accordance with the ESS guidelines^23^, these were applied as prior weights in the model estimation rather than as a covariate. All estimated prevalences and their associated statistical tests were age-standardized by holding the continuous age variable constant at a fixed reference age of 70 years when calculating the marginal predictions. The proportion of missing values of the variables used was very low (< 1%). Thus, we refrained from imputing missing data and proceeded with a complete-case analysis.

### Sensitivity analysis

To account for potential heterogeneity in the distribution of age and education across countries and over time, we conducted several sensitivity checks to verify the robustness of our main findings. First, we repeated the trend analyses of the prevalence using the identical logistic multilevel regression approach, but stratified by three age groups (60–69 (total in the 11 waves n = 48,569), 70–79 (n = 33,994), and 80 years and older (n = 15,737); please see Supplementary Figures S1–S6). As a further robustness check, we analysed absolute and relative inequalities between the low- and high-education groups in the most recent ESS wave (2023). Absolute inequalities were measured as the risk difference in percentage points, while relative inequalities were calculated as the rate ratio of disability prevalences between these two groups (Supplementary Tables S1 and S2). In terms of time trends, we additionally calculated both the age-standardized Slope and Relative Index of Inequality (SII and RII^24^). Because they are based on the relative rank of the educational groups, these indices correct for varying population sizes across the educational strata over time (Supplementary Figures S7 and S8). Finally, to formally test whether the changes in educational inequalities in disability over time significantly differed between women and men, we fitted an additional logistic multilevel model using the combined dataset. This model included a three-way interaction term (survey wave × education × gender) to allow for pairwise comparisons of the gender-specific inequality trends within each country (Supplementary Table S3).

All analyses were conducted using R statistics^25^, including packages modelbased^26^, glmmTMB^27^ and ggplot2^28^. Data is available from the ESS website. All R scripts used for data analysis are stored at the zenodo repository (10.5281/zenodo.17234405).

## Results

Sample description (i.e., sample size of individuals aged 60 years and older, age, proportion of women, prevalence of low education and of disability) for the 15 European countries is shown in Table 1. For this description, data of the 11 waves were pooled. Average prevalence of disability among older adults between 2002 and 2023 was highest in Slovenia (56%) and lowest in Ireland (28%).

**Table 1:** Sample description (pooled data weighted by respondent-level post-stratification weights), pooled values and range across waves by country (European Social Survey, respondents aged 60 + years, waves 1–11, 2002–2023).

Country	n (range)	Age, mean (range)	Women, % (range)	Low education, % (range)	Disability, % (range)
BE	5,285 (409–551)	71 (71–71)	53 (49–56)	54 (39–69)	39 (34–42)
CH	5,433 (416–623)	71 (70–72)	54 (53–56)	27 (18–33)	32 (28–37)
DE	11,480 (773–2,935)	71 (70–71)	54 (52–56)	26 (20–35)	50 (45–52)
ES	6,089 (406–728)	71 (71–72)	55 (52–57)	79 (64–90)	34 (24–41)
FI	7,143 (481–796)	71 (70–72)	56 (53–59)	47 (30–68)	48 (44–53)
FR	6,933 (422–825)	71 (71–72)	55 (53–56)	56 (40–73)	38 (34–43)
GB	7,994 (526–865)	71 (70–72)	53 (52–55)	55 (33–80)	42 (40–45)
HU	6,007 (401–706)	71 (70–72)	59 (58–61)	42 (25–65)	53 (45–59)
IE	7,541 (439–894)	70 (69–71)	51 (49–55)	56 (41–69)	28 (24–34)
NL	6,436 (450–681)	70 (69–71)	52 (50–54)	50 (44–58)	39 (36–41)
NO	4,416 (365–440)	71 (70–71)	52 (47–56)	33 (24–47)	38 (34–43)
PL	5,067 (315–681)	70 (69–71)	58 (54–60)	53 (41–63)	54 (42–63)
PT	7,588 (392–1,020)	71 (70–72)	55 (53–57)	88 (77–96)	34 (28–40)
SE	6,543 (496–938)	71 (69–73)	52 (49–54)	37 (25–48)	41 (36–46)
SI	4,345 (359–431)	71 (70–71)	57 (52–61)	37 (26–50)	56 (50–63)
Overall	98,300 (7,611–11,407)	71 (70–71)	54 (53–56)	48 (36–60)	42 (40–45)

BE, Belgium; CH, Switzerland; DE, Germany; ES, Spain; FI, Finland; FR, France; GB, Great Britain; HU, Hungary; IE, Ireland; NL, the Netherlands; NO, Norway; PL, Poland; PT, Portugal; SE, Sweden; and Sl, Slovenia.

In the latest wave of the ESS (2023), there was a general pattern of an educational gradient in the prevalence of disability regarding the three educational groups among men and women aged 60 + years in the 15 European countries (Table 2). Differences were significant (*p* < 0.05) in eight countries. Significant educational inequalities among both, men and women emerged in Belgium, Germany, France, Great Britain, and Hungary. Table S1 and S2 (please see Supplementary Information) show the absolute and relative educational inequalities in disability for men and women in the 15 countries. Absolute inequalities (rate differences in % points) between low and high education range from 0 to 45.2% among men and from − 1.3 to 32.5% among women. Range of relative inequalities (rate ratios) was from 1.00 to 9.83 among men and from 0.96 to 2.69 among women.

**Table 2:** Prevalence of disability (in %, and 95% confidence intervals) by educational level, gender, and country (European Social Survey, wave 11, 2023, respondents aged 60 + years, weighted data, age-standardized).

Country (n men; n women)	Men				Women			
	Education			p*	Education			p*
	Low	Mid	High		Low	Mid	High	
BE (230; 266)	49.0 (39.17, 58.96)	38.1 (28.01, 49.24)	27.1 (18.55, 37.85)	**0.022**	53.5 (43.92, 62.92)	33.5 (24.52, 43.80)	35.6 (25.49, 47.18)	**0.019**
CH (205; 229)	46.4 (28.15, 65.75)	34.4 (26.78, 42.86)	23.0 (13.38, 36.70)	0.116	39.6 (27.22, 53.56)	34.0 (26.58, 42.32)	27.5 (15.38, 44.08)	0.655
DE (399; 437)	72.8 (60.99, 82.14)	52.5 (46.85, 58.08)	27.6 (18.37, 39.27)	** < 0.001**	54.7 (44.98, 64.10)	40.5 (34.39, 46.83)	30.3 (18.29, 45.72)	**0.019**
ES (268; 292)	22.7 (16.89, 29.78)	17.4 (9.25, 30.26)	11.6 (5.90, 21.69)	0.137	28.3 (22.17, 35.36)	24.0 (13.84, 38.18)	20.0 (10.75, 34.24)	0.469
FI (269; 303)	57.7 (47.09, 67.60)	49.0 (41.52, 56.53)	33.0 (21.83, 46.45)	**0.028**	51.5 (40.82, 62.13)	53.1 (45.47, 60.58)	37.7 (26.09, 50.96)	0.229
FR (261; 311)	46.8 (36.74, 57.14)	28.2 (21.47, 36.03)	21.8 (12.31, 35.53)	**0.009**	43.9 (35.49, 52.64)	29.4 (22.41, 37.41)	22.9 (11.48, 40.41)	**0.034**
GB (252; 278)	49.7 (40.43, 58.95)	43.9 (35.03, 53.09)	28.6 (19.64, 39.54)	**0.010**	53.7 (44.93, 62.34)	42.2 (34.10, 50.77)	25.3 (16.36, 37.06)	** < 0.001**
HU (270; 377)	75.0 (57.53, 86.93)	46.6 (38.38, 54.91)	39.4 (26.00, 54.52)	**0.004**	47.6 (38.13, 57.16)	46.6 (40.03, 53.24)	26.3 (17.02, 38.20)	** < 0.001**
IE (226; 237)	30.6 (24.16, 37.87)	27.3 (20.40, 35.39)	21.0 (13.39, 31.43)	0.338	37.6 (30.07, 45.77)	23.6 (17.75, 30.77)	21.5 (13.80, 31.79)	**0.017**
NL (267; 276)	41.4 (32.65, 50.70)	40.0 (31.14, 49.56)	25.1 (16.70, 35.89)	0.070	51.8 (43.16, 60.34)	38.5 (29.50, 48.40)	19.3 (10.48, 32.72)	** < 0.001**
NO (191; 201)	51.6 (38.29, 64.61)	34.3 (25.95, 43.72)	21.9 (12.53, 35.52)	**0.011**	58.5 (44.67, 71.14)	48.5 (37.97, 59.24)	39.2 (26.36, 53.77)	0.252
PL (265; 314)	53.1 (43.24, 62.73)	36.1 (25.93, 47.69)	32.2 (19.62, 48.08)	0.052	46.1 (35.80, 56.77)	43.5 (34.39, 52.97)	25.1 (14.06, 40.79)	0.091
PT (206; 251)	31.4 (24.65, 39.04)	20.5 (9.47, 38.82)	3.2 (0.32, 25.35)	** < 0.001**	30.6 (24.94, 36.86)	24.6 (12.84, 41.86)	31.8 (19.05, 48.11)	0.638
SE (194; 208)	38.6 (27.37, 51.22)	40.8 (33.01, 49.06)	38.7 (26.89, 51.96)	0.948	52.8 (38.48, 66.68)	46.7 (38.00, 55.64)	36.4 (24.61, 50.01)	0.384
SI (194; 210)	59.4 (41.54, 75.01)	54.8 (45.79, 63.49)	36.5 (21.09, 55.24)	0.242	59.0 (47.15, 69.85)	53.7 (44.31, 62.75)	29.4 (15.44, 48.80)	**0.040**
*All countries (3,697; 4,190)*	43.5 (40.93, 46.13)	41.3 (39.11, 43.50)	26.6 (23.75, 29.64)	** < 0.001**	44.5 (42.17, 46.93)	40.6 (38.50, 42.82)	29.0 (25.93, 32.38)	** < 0.001**

*Significance of global omnibus test; BE, Belgium; CH, Switzerland; DE, Germany; ES, Spain; FI, Finland; FR, France; GB, Great Britain; HU, Hungary; IE, Ireland; NL, the Netherlands; NO, Norway; PL, Poland; PT, Portugal; SE, Sweden; and Sl, Slovenia.

Significant values are in bold.

**Fig. 1:**
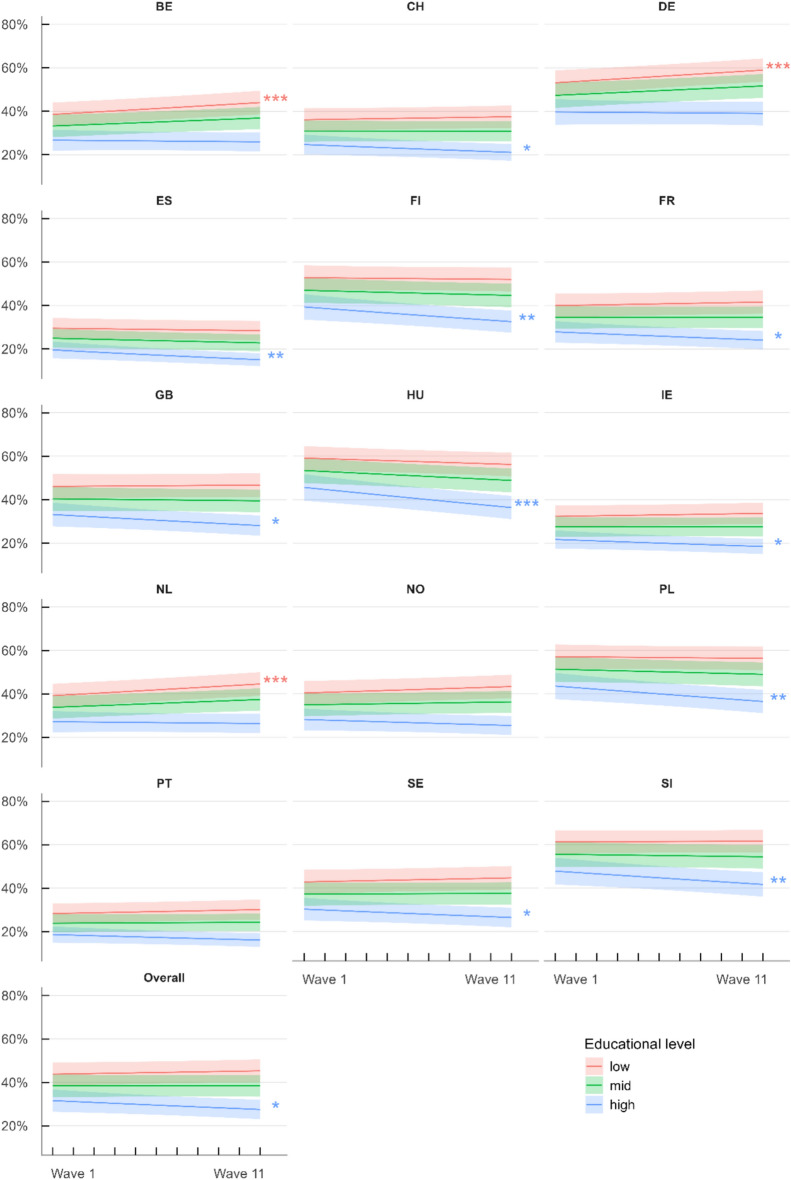
Trends in prevalence of disability by education level and country; *men*; age-standardized; significance of trend for each education level (*p < 0.05; **p < 0.01; ***p < 0.001) (European Social Survey, respondents aged 60 + years, waves 1–11, 2002–2023).

Figure 1 shows the trends in prevalence of disability between 2002 and 2023 (11 waves of the ESS) by educational level among men for the 15 countries. In Belgium, Germany, and the Netherlands, the prevalence of disability significantly increased among men with a low education. There was a significant decrease among highly educated men in ten countries (Switzerland, Spain, Finland, France, Great Britain, Hungary, Ireland, Poland, Sweden, and Slovenia).

**Fig. 2:**
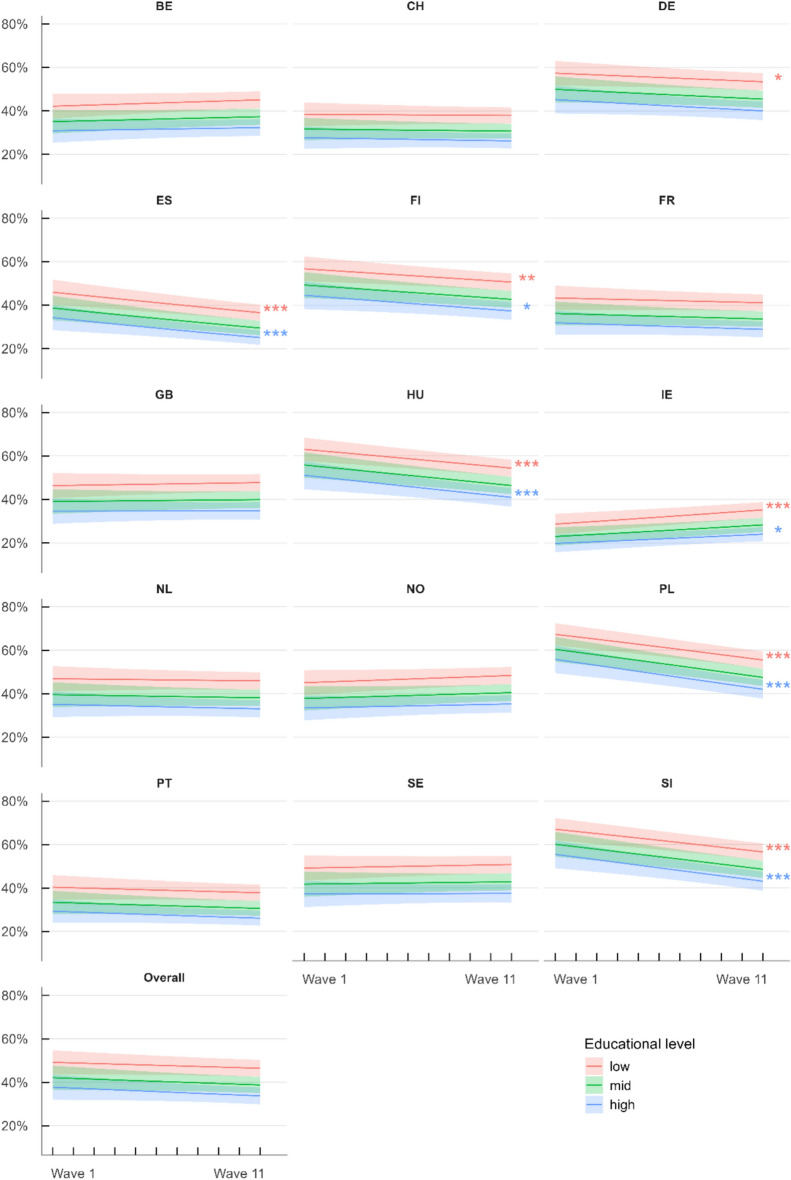
Trends in prevalence of disability by education level and country; *women*; age-standardized; significance of trend for each education level (*p < 0.05; **p < 0.01; ***p < 0.001) (European Social Survey, respondents aged 60 + years, waves 1–11, 2002–2023).

Figure 2 shows the respective trends for women. There were significant decreases of the prevalence of disability among high and low educated women in Spain, Finland, Hungary, Poland, and Slovenia. Contrary to this trend, women with a high and a low education were affected by an increase of disability in Ireland. Figures S1 to S6 (please see Supplementary Information) show variations in these trends according to age (60–69 years, 70–79 years, and 80 + years) for men and women. Age difference were more pronounced among men than among women.

**Fig. 3:**
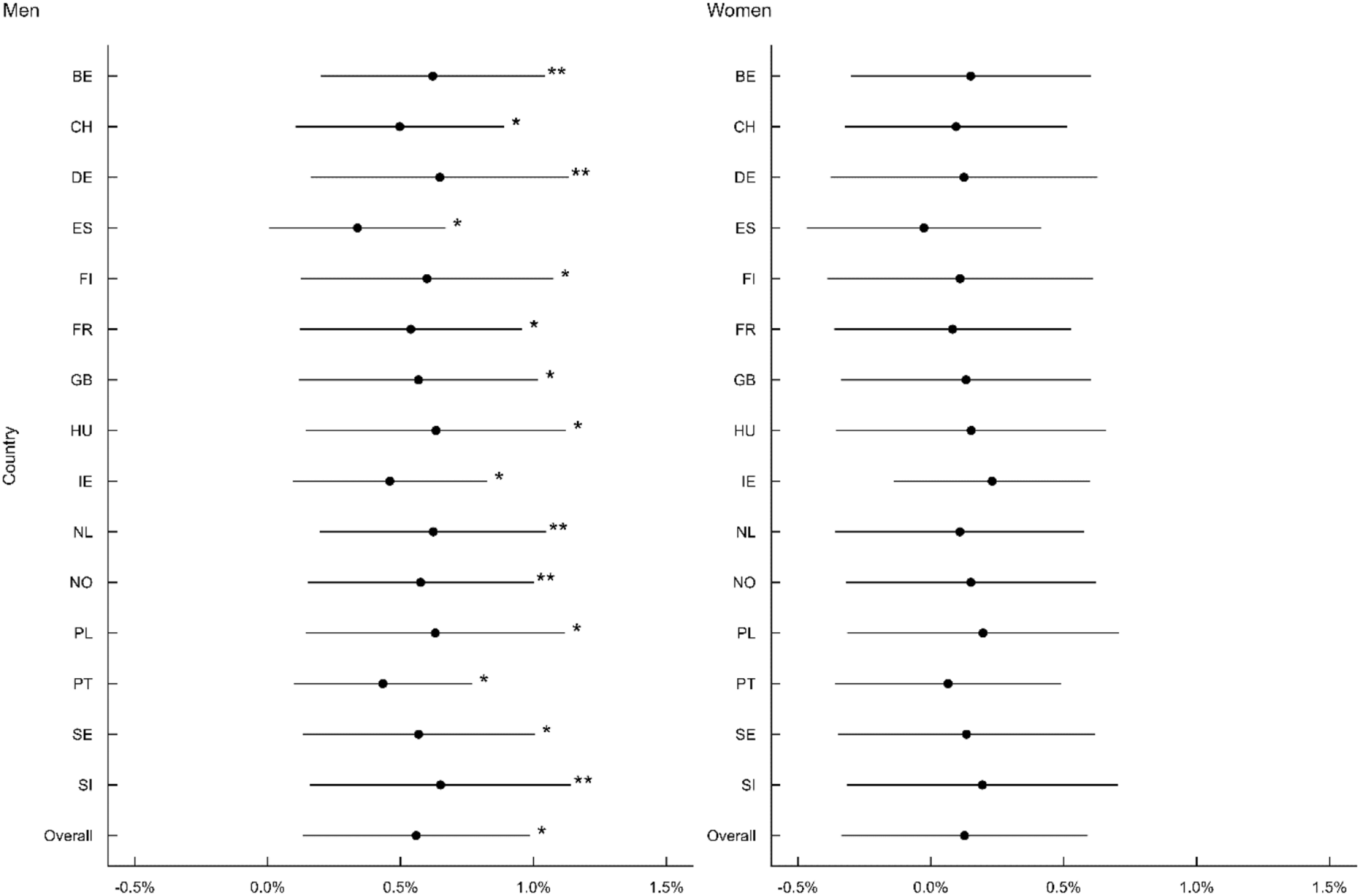
Average change of educational inequalities in disability by country and gender per wave (in % points); age-standardized; significance of changes (*p < 0.05; **p < 0.01; ***p < 0.001) (European Social Survey, respondents aged 60 + years, waves 1–11, 2002–2023).

To illustrate trends in educational inequalities in disability, Fig. 3 shows the average change of the differences in the prevalence between low and high educational groups per ESS wave among men (left) and women (right). Among men, there is a significant increase of educational inequalities over time in all European countries under study. Overall, inequalities in the prevalence between the two groups increased by more than 0.5% per wave. Among women, there is a trend for an increase, but the average change is not significant in any of the included European countries. Table S3 (please see Supplementary Information) indicates that the gender differences were not significant in any country. Figures S7 and S8 show the trends among men and women when the Slope Index of Inequality (SII) and the Relative Index of Inequality (RII) are used. Overall, absolute and relative educational inequalities tend to increase among men (please see black lines for total trends), while this trend was less pronounced among women, indicating robustness of our main finding. However, considerable variations across countries emerged.

## Discussion

### Summary and Interpretation

Based on the 11 available waves of the ESS (2002 to 2023), we examined the magnitude and trends of educational inequalities in disability among older men and women (≥ 60 years) in 15 European countries. In the latest wave of the ESS (2023), there was an educational gradient in the prevalence of disability in the 15 European countries. However, there were country differences regarding the significance of inequalities. Significant educational inequalities among both, men and women were observed in Belgium, Germany, France, Great Britain, and Hungary. In terms of time trends among men, disability significantly declined in high education groups in ten countries. In two countries, there were no significant changes among the education groups between 2002 and 2023, while in three countries prevalence increased among those with low education. Trends in prevalence were quite different among women. There was a significant decline among low as well as high education groups in five countries and prevalences were stable in eight countries. Overall, among men, there was a significant increase of educational inequalities in disability between the two groups over time in the European countries, while this trend was not significant among women.

Similar to previous studies^2,14^, we found educational inequalities in disability among the aged in a number of European countries in 2023. These inequalities were significant among both, men and women in five of the 15 countries under study (Belgium, Germany, France, Great Britain, and Hungary). Germany and France also showed significant income-related inequalities in disability among older men and women in 2014 in a study that used ESS data as well^29^. Overall, our analyses of the most recent ESS data revealed that the magnitude of educational inequalities was similar among men and women and that the association between education and disability showed the pattern of a gradient. These results align with prior research showing that education among other social factors may promote healthy aging, longevity, and well-being in old age^30^.

Trends in the prevalence of disability differed according to gender. Among men, in the majority of the countries under study (10 out of 15), disability significantly declined in high education groups only. Among women, trends in the prevalence of disability were similar in the educational groups in almost all countries (except Germany), i.e. disability either remained stable (in eight countries), declined (in five countries), or increased (in Ireland). Previous studies on time trends in disability mostly did not consider (educational) inequalities. For example, Jagger et al.^31^ explored trends in disability-free life expectancy among men and women aged 65 years and older in England and found increases in mild limitations between 1991 and 2011. In an international study analysing trends in disability prevalence among older adults between 2004 and 2014^12^, there was a decrease in some European countries (e.g. Poland, Sweden, and Denmark). However, in other countries (e.g. Germany, Belgium, and Czechia), prevalence increased in the same period. In the study, gender differences were not reported.

Furthermore, our study revealed that there was a significant increase of inequalities in disability between low and high educational groups from 2002 to 2023 in all 15 European countries among older men, while this trend was not significant in any country among older women. Findings discussed in the previous paragraph indicate that the increase of inequalities among men was accompanied by a decline in disability among the high-educated group. The stability of inequalities among women coincided with the similar trends in the prevalence of disability in the different educational groups. A few studies analysed trends of educational inequalities in disability in Europe. However, none of these is fully comparable with our study. Based on analyses in 26 European countries Rubio-Valverde et al.^17^ found that inequalities tended to increase between 2002 and 2017 among men and women aged 30 to 79 years. In a study by Wilkie et al.^13^, there was no clear trend regarding educational inequalities among people aged 65 years and older in 11 high income European countries comparing two time points (2004 and 2018). However, trends were not reported separately for men and women. Overall, the present study contributes to the field by showing diverging recent trends of educational inequalities in disability for older men and women in a number of European countries.

### Limitations

Some limitations have to be considered when interpreting and evaluating the presented results. First, the cross-sectional design of the ESS does not allow any causal interpretations. Moreover, due to the study design, we were not able to distinguish age, period, and cohort effects. Second, although the ESS has high methodological standards, non-response and cross-national differences in response rates may affect the findings^32^. While data was weighted to reduce the impact of low response rates^23^, we cannot rule out a selection bias due to non-response. Sample sizes per wave were quite small in some countries which also has to be considered when interpreting findings. Third, until wave 10 (2020) of the survey, all countries were required to conduct face-to-face data collection. However, the COVID pandemic made face-to-face data collection difficult to implement. A total of nine countries switched to a self-completion approach, while 22 countries were able to use the usual face-to-face approach^18^. It is possible that this switch in some countries had an effect on the reporting of disability and thus, on the results presented. Fourth, the indicator of disability was based on a single-item question only. Although this simple measure does not ask for difficulties with different activities, it is considered a valuable disability indicator for population health monitoring in Europe, also referred to as the Global Activity Limitation Indicator (GALI)^17^. It has been used in previous international comparisons of social inequalities in disability^16,17,29^ and was found to have a reasonable reliability^33^. However, the wording of the indicator used in all waves of the ESS, slightly differed from other European surveys (e.g. it does not have the reference to the 6-month time frame). Moreover, the indicator is based on self-reports and we did not distinguish between levels of disability. Fifth, in terms of the ISCED categories used, it has to be considered that the prevalence of low education was different in the countries and varied over time. Thus, this category captures different social positions in different countries and times. While our sensitivity analyses using RII and SII account for these distributional shifts, the models used for the main results may still be subject to residual confounding by unmeasured factors such as life-course conditions. Finally, we did not include structural and cultural factors (e.g. welfare states, cultural norms, health behaviour) in the analyses that may help to interpret the presented country differences in educational inequalities.

## Conclusions

Educational inequalities in disability appeared to have increased or at least persisted since the beginning of the 2000s in Europe. Especially among men, improvements in functional ability in older age (which is an indicator of healthy aging) were found to be restricted to highly educated groups. The mechanisms behind these inequalities in disability are not yet fully understood. Future studies should investigate to what extent behavioural, psychosocial, and material factors contribute to their explanation. A better understanding of these explanatory factors is needed to develop and implement interventions for a reduction of inequalities in disability in aging societies.

## Supplementary Information

Supplementary Information.

## Data Availability

The dataset used is publicly available from https://www.europeansocialsurvey.org.
