# Supplementary material for: Educational inequalities in disability among older adults: a trend analysis in 15 European countries (2002 to 2023)
Source: Sci Rep. 2026 Jul 20;16:22663. doi: 10.1038/s41598-026-62524-0 (PMC13385872; doi:10.1038/s41598-026-62524-0)

Supplementary information

for the article "Educational inequalities in disability among older adults - a trend analysis in 15 European countries (2002 to 2023)"

Table S1: Absolute inequalities in disability prevalence (in % between low and high education) by gender and country (European Social Survey, wave 11, 2023, respondents aged 60+ years, weighted data, age-standardized)

	Men			Women		
Country	Difference in % points	95% confidence intervals	p	Difference in % points	95% confidence intervals	p
BE	21.9	(8.04, 35.74)	0.002	17.9	(3.33, 32.57)	0.016
CH	23.4	(0.83, 46.00)	0.042	12.2	(-7.78, 32.15)	0.230
DE	45.2	(30.23, 60.22)	< 0.001	24.4	(7.45, 41.41)	0.005
ES	11.1	(1.07, 21.04)	0.030	8.3	(-5.21, 21.77)	0.228
FI	24.7	(8.63, 40.75)	0.003	13.8	(-2.92, 30.57)	0.105
FR	25.0	(9.40, 40.69)	0.002	21.0	(3.98, 38.05)	0.016
GB	21.1	(7.58, 34.66)	0.002	28.4	(14.77, 42.05)	< 0.001
HU	35.7	(14.79, 56.53)	< 0.001	21.3	(6.81, 35.80)	0.004
IE	9.6	(-1.78, 20.90)	0.098	16.1	(4.02, 28.25)	0.009
NL	16.3	(3.04, 29.54)	0.016	32.5	(18.31, 46.77)	< 0.001
NO	29.6	(11.94, 47.32)	0.001	19.3	(-0.55, 39.16)	0.057
PL	20.9	(3.28, 38.49)	0.020	21.0	(3.79, 38.17)	0.017
PT	28.2	(18.02, 38.40)	< 0.001	-1.3	(-17.39, 14.85)	0.877
SE	0.0	(-17.40, 17.29)	0.995	16.4	(-2.78, 35.65)	0.093
SI	22.9	(-1.93, 47.68)	0.070	29.5	(8.85, 50.22)	0.005
Overall	16.9	(13.02, 20.82)	< 0.001	15.5	(11.47, 19.51)	< 0.001

BE=Belgium, CH=Switzerland, DE=Germany, ES=Spain, FI=Finland, FR=France, GB=Great Britain, HU=Hungary, IE=Ireland, NL=the Netherlands, NO=Norway, PL=Poland, PT=Portugal, SE=Sweden, and Sl=Slovenia

Table S2: Relative inequalities in disability prevalence (rate ratios for low and high education) by gender and country (European Social Survey, wave 11, 2023, respondents aged 60+ years, weighted data, age-standardized)

	Men			Women		
Country	Rate ratio	95% confidence intervals	p	Rate ratio	95% confidence intervals	p
BE	1.81	(1.07, 2.55)	0.033	1.50	(0.97, 2.04)	0.066
CH	2.02	(0.70, 3.33)	0.128	1.44	(0.53, 2.36)	0.340
DE	2.64	(1.56, 3.72)	0.003	1.81	(0.91, 2.70)	0.076
ES	1.95	(0.56, 3.34)	0.181	1.41	(0.52, 2.31)	0.363
FI	1.75	(1.02, 2.48)	0.044	1.37	(0.82, 1.91)	0.186
FR	2.15	(0.90, 3.40)	0.071	1.92	(0.63, 3.20)	0.160
GB	1.74	(1.05, 2.43)	0.035	2.12	(1.18, 3.06)	0.019
HU	1.91	(1.10, 2.71)	0.027	1.81	(0.98, 2.64)	0.055
IE	1.45	(0.75, 2.16)	0.205	1.75	(0.92, 2.58)	0.075
NL	1.65	(0.92, 2.38)	0.081	2.69	(1.07, 4.31)	0.041
NO	2.35	(0.97, 3.73)	0.055	1.49	(0.85, 2.14)	0.135
PL	1.65	(0.84, 2.45)	0.114	1.83	(0.76, 2.91)	0.127
PT	9.83	(-12.46, 32.13)	0.436	0.96	(0.47, 1.45)	0.873
SE	1.00	(0.55, 1.45)	0.995	1.45	(0.81, 2.10)	0.169
SI	1.63	(0.70, 2.55)	0.182	2.00	(0.77, 3.24)	0.110
Overall	1.64	(1.43, 1.84)	< 0.001	1.53	(1.34, 1.72)	<0 .001

BE=Belgium, CH=Switzerland, DE=Germany, ES=Spain, FI=Finland, FR=France, GB=Great Britain, HU=Hungary, IE=Ireland, NL=the Netherlands, NO=Norway, PL=Poland, PT=Portugal, SE=Sweden, and Sl=Slovenia

Table S3: Difference in average change of educational inequalities in disability per wave by country between men and women (in % points) (European Social Survey, respondents aged 60+ years, age-standardized, waves 1 – 11, 2002 – 2023, based on 3-way-interaction model)

Country	Difference (in %-point)	95% confidence interval	p
BE	0.53	(-0.08, 1.13)	0.088
CH	0.46	(-0.09, 1.02)	0.104
DE	0.55	(-0.13, 1.23)	0.115
ES	0.42	(-0.12, 0.96)	0.131
FI	0.51	(-0.16, 1.19)	0.137
FR	0.49	(-0.11, 1.08)	0.110
GB	0.52	(-0.11, 1.16)	0.108
HU	0.50	(-0.19, 1.20)	0.153
IE	0.44	(-0.06, 0.95)	0.084
NL	0.52	(-0.10, 1.14)	0.098
NO	0.53	(-0.09, 1.15)	0.096
PL	0.50	(-0.19, 1.20)	0.156
PT	0.44	(-0.09, 0.97)	0.103
SE	0.53	(-0.11, 1.17)	0.105
SI	0.51	(-0.18, 1.21)	0.148
Overall	0.50	(-0.12, 1.12)	0.115

BE=Belgium, CH=Switzerland, DE=Germany, ES=Spain, FI=Finland, FR=France, GB=Great Britain, HU=Hungary, IE=Ireland, NL=the Netherlands, NO=Norway, PL=Poland, PT=Portugal, SE=Sweden, and Sl=Slovenia

Figure S1: Trends in prevalence of disability by education level and country; age-standardized; significance of trend for each education level (*p<0.05; **p<0.01; ***p<0.001) (European Social Survey, respondents aged 60+ years, waves 1 – 11, 2002 – 2023); **men, 60-69 years**

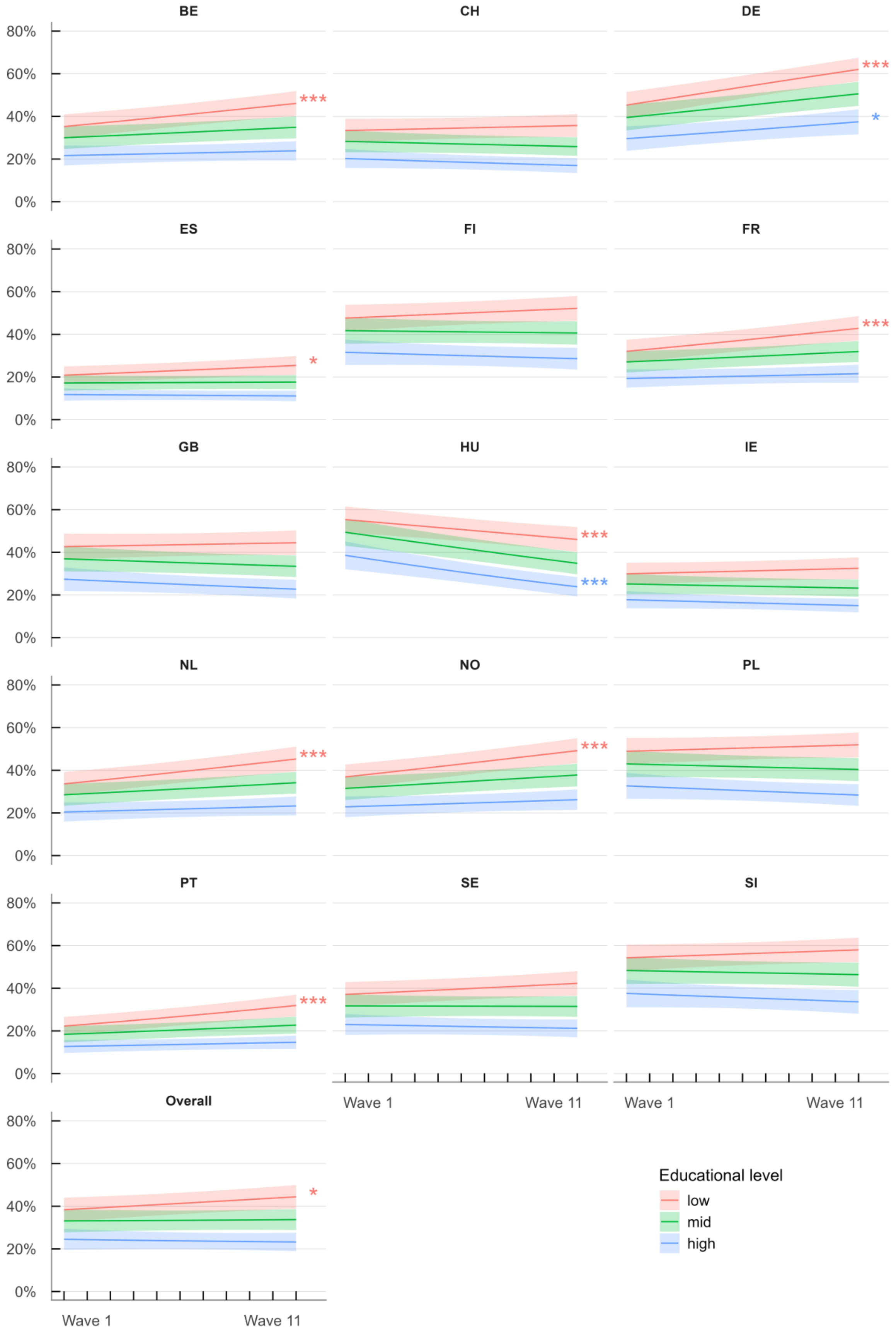

Figure S2: Trends in prevalence of disability by education level and country; age-standardized; significance of trend for each education level (*p<0.05; **p<0.01; ***p<0.001) (European Social Survey, respondents aged 60+ years, waves 1 – 11, 2002 – 2023); **women, 60-69 years**

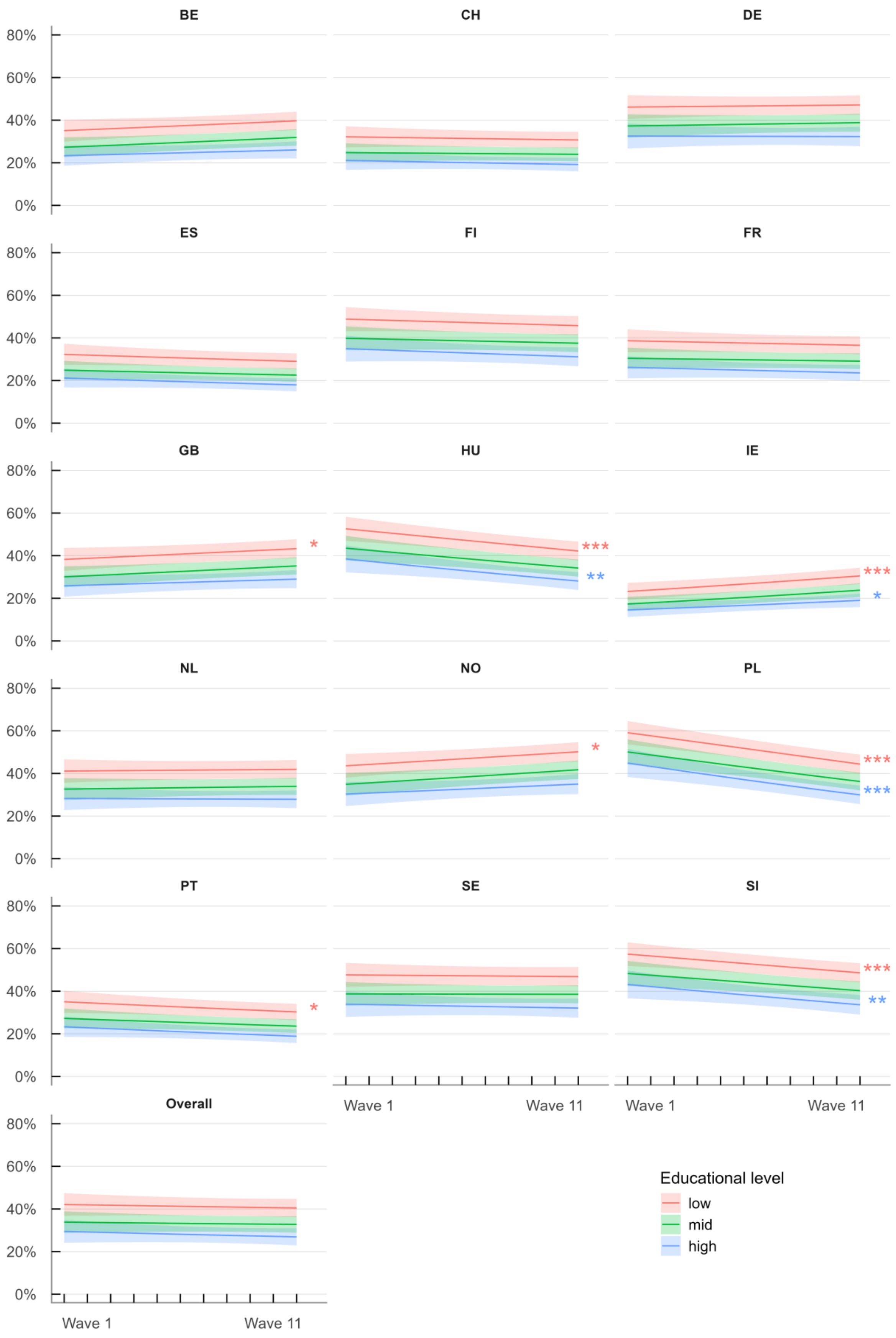

Figure S3: Trends in prevalence of disability by education level and country; age-standardized; significance of trend for each education level (*p<0.05; **p<0.01; ***p<0.001) (European Social Survey, respondents aged 60+ years, waves 1 – 11, 2002 – 2023); **men, 70-79 years**

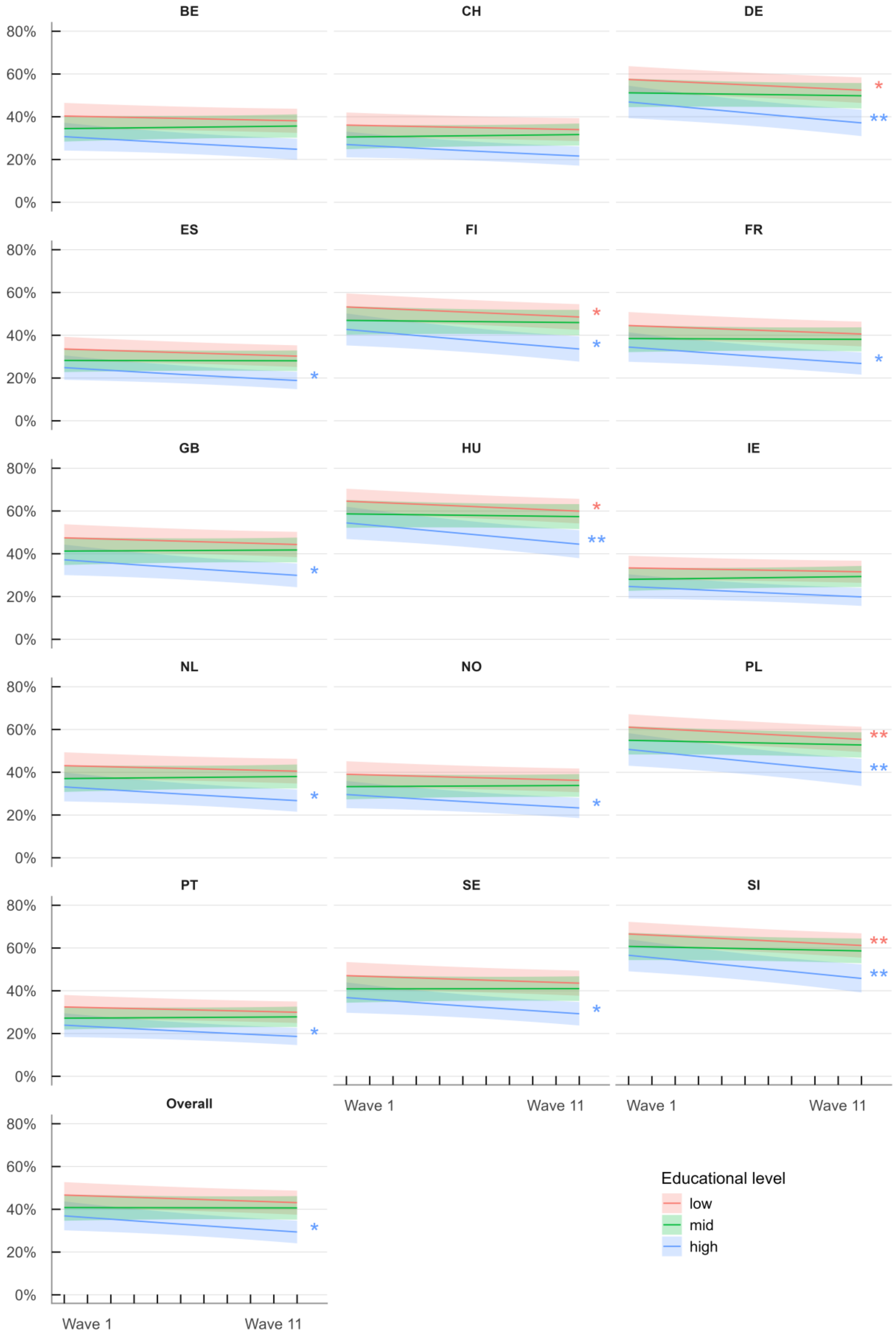

Figure S4: Trends in prevalence of disability by education level and country; age-standardized; significance of trend for each education level (*p<0.05; **p<0.01; ***p<0.001) (European Social Survey, respondents aged 60+ years, waves 1 – 11, 2002 – 2023); **women, 70-79 years**

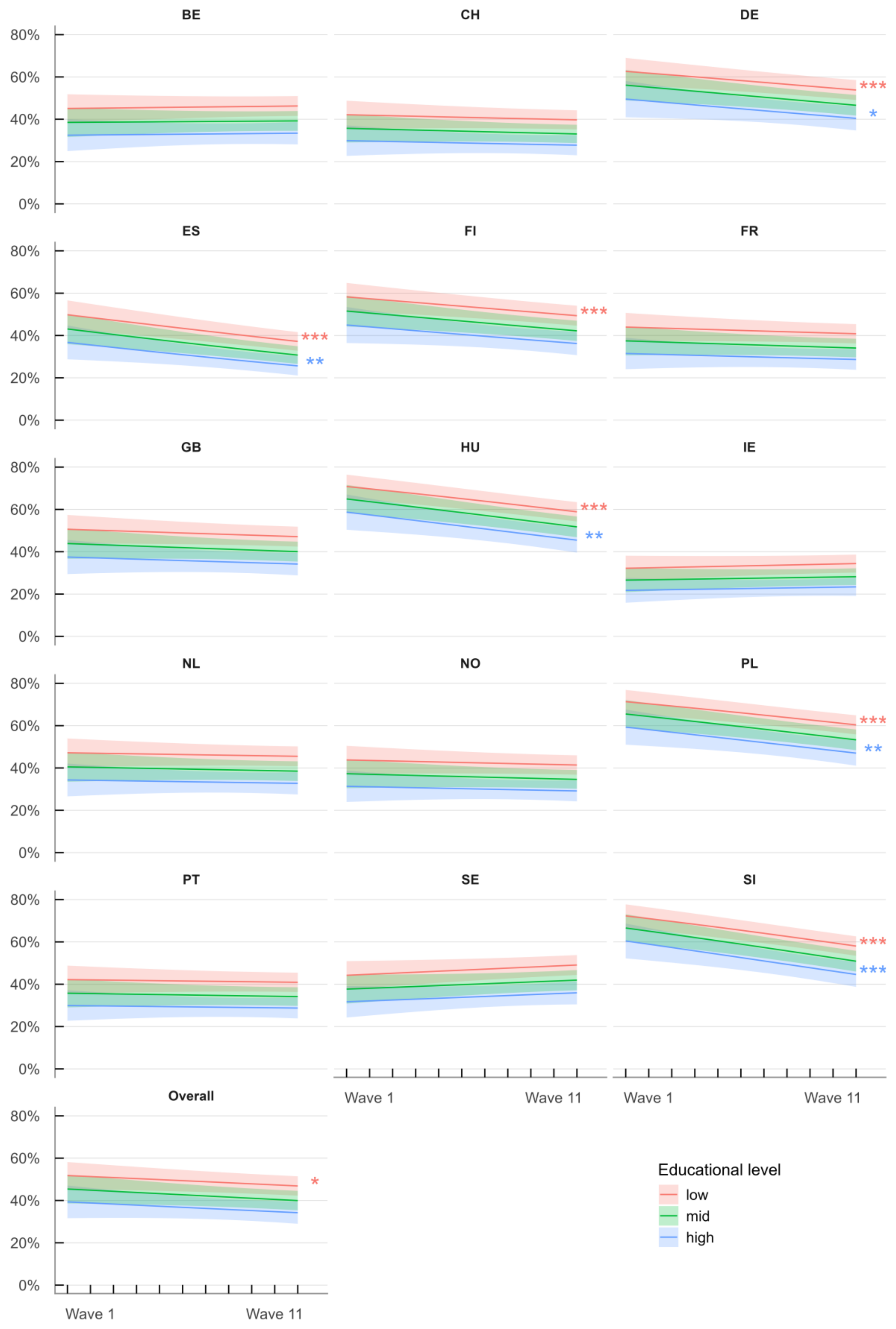

Figure S5: Trends in prevalence of disability by education level and country; age-standardized; significance of trend for each education level (*p<0.05; **p<0.01; ***p<0.001) (European Social Survey, respondents aged 60+ years, waves 1 – 11, 2002 – 2023); **men, 80+ years**

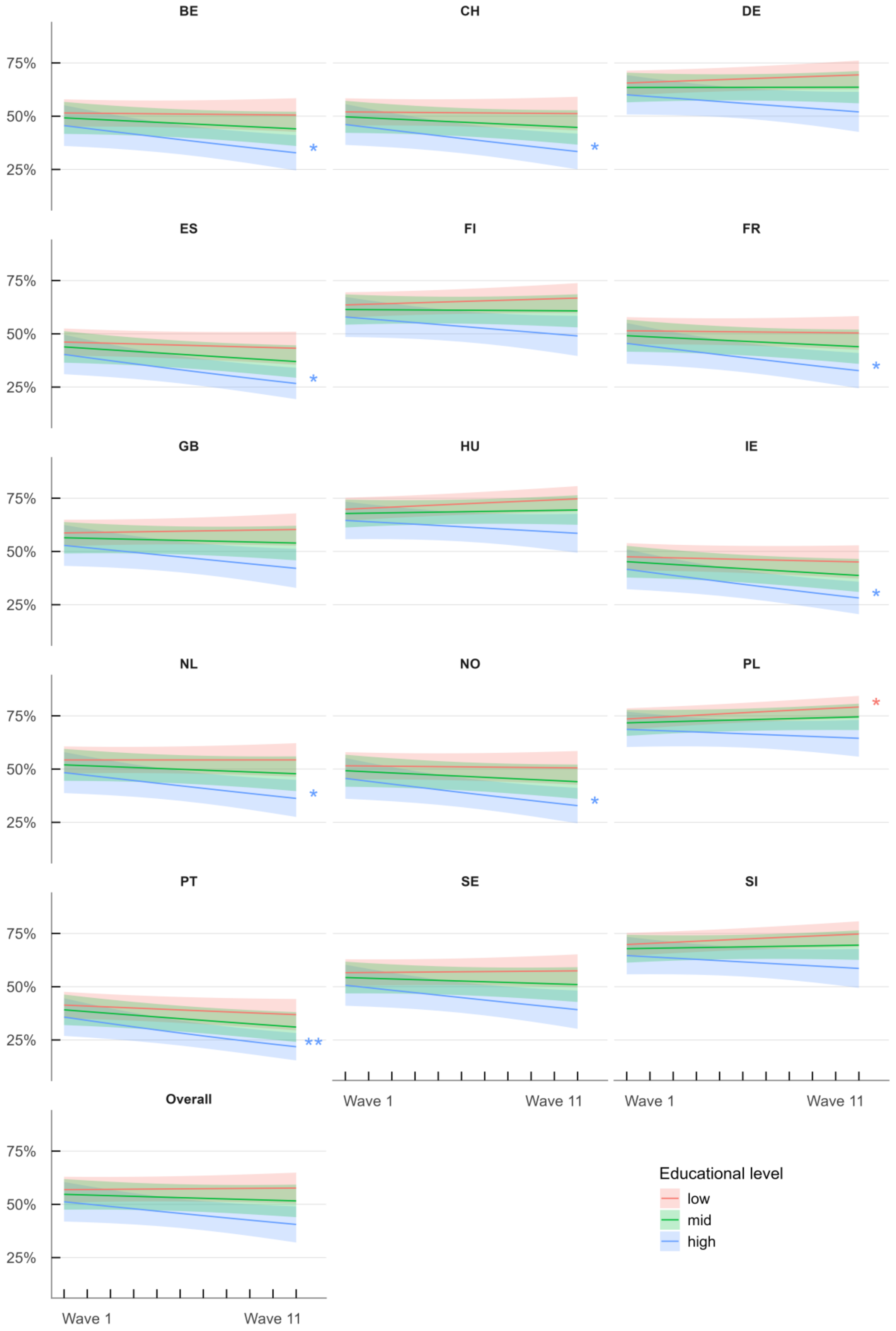

Figure S6: Trends in prevalence of disability by education level and country; age-standardized; significance of trend for each education level (*p<0.05; **p<0.01; ***p<0.001) (European Social Survey, respondents aged 60+ years, waves 1 – 11, 2002 – 2023); **women, 80+ years**

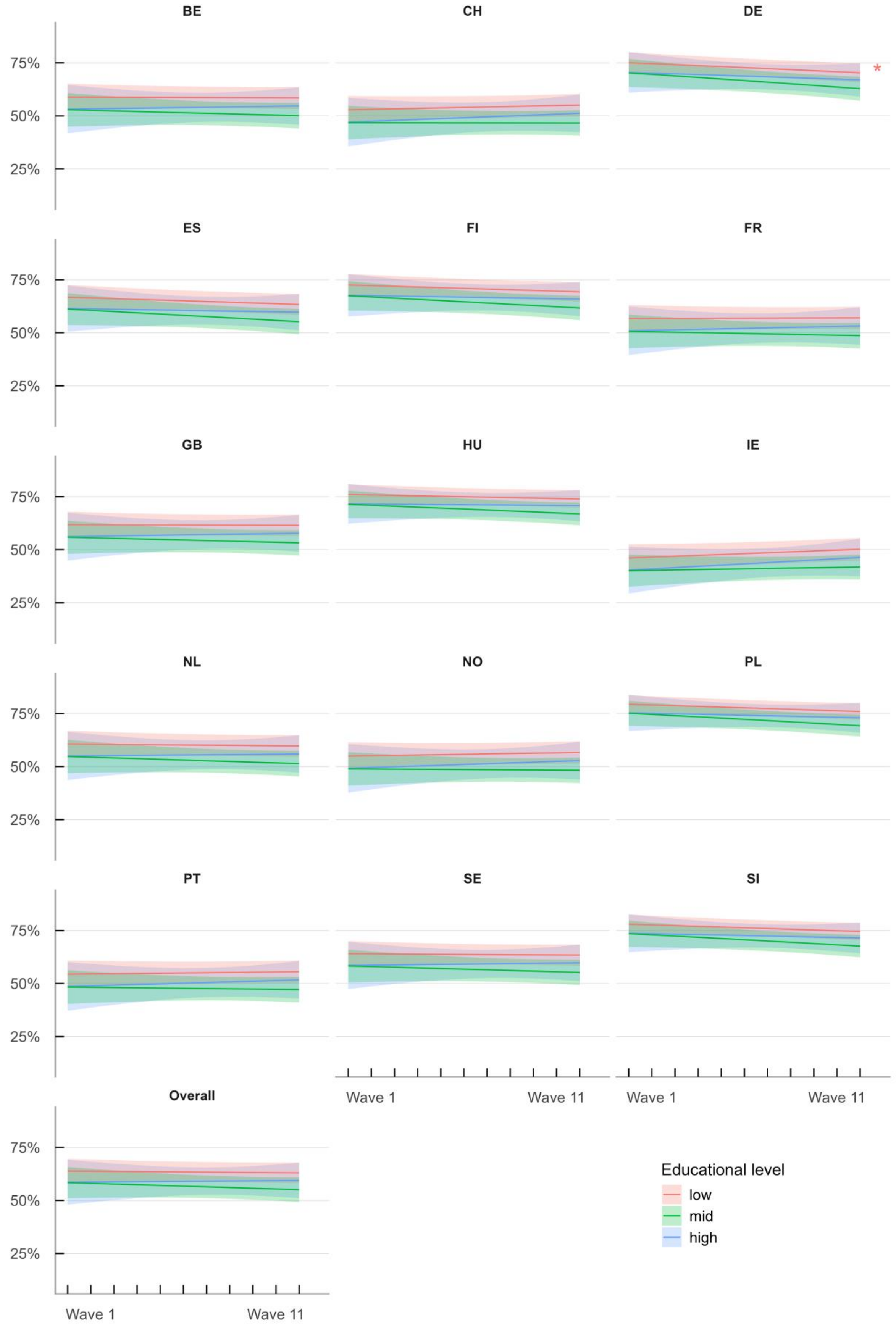

Figure S7: Slope Index of Inequality (SII) for disability by country and gender across all waves; age-standardized (European Social Survey, respondents aged 60+ years, waves 1 – 11, 2002 – 2023); higher SII values indicate greater inequality between education groups

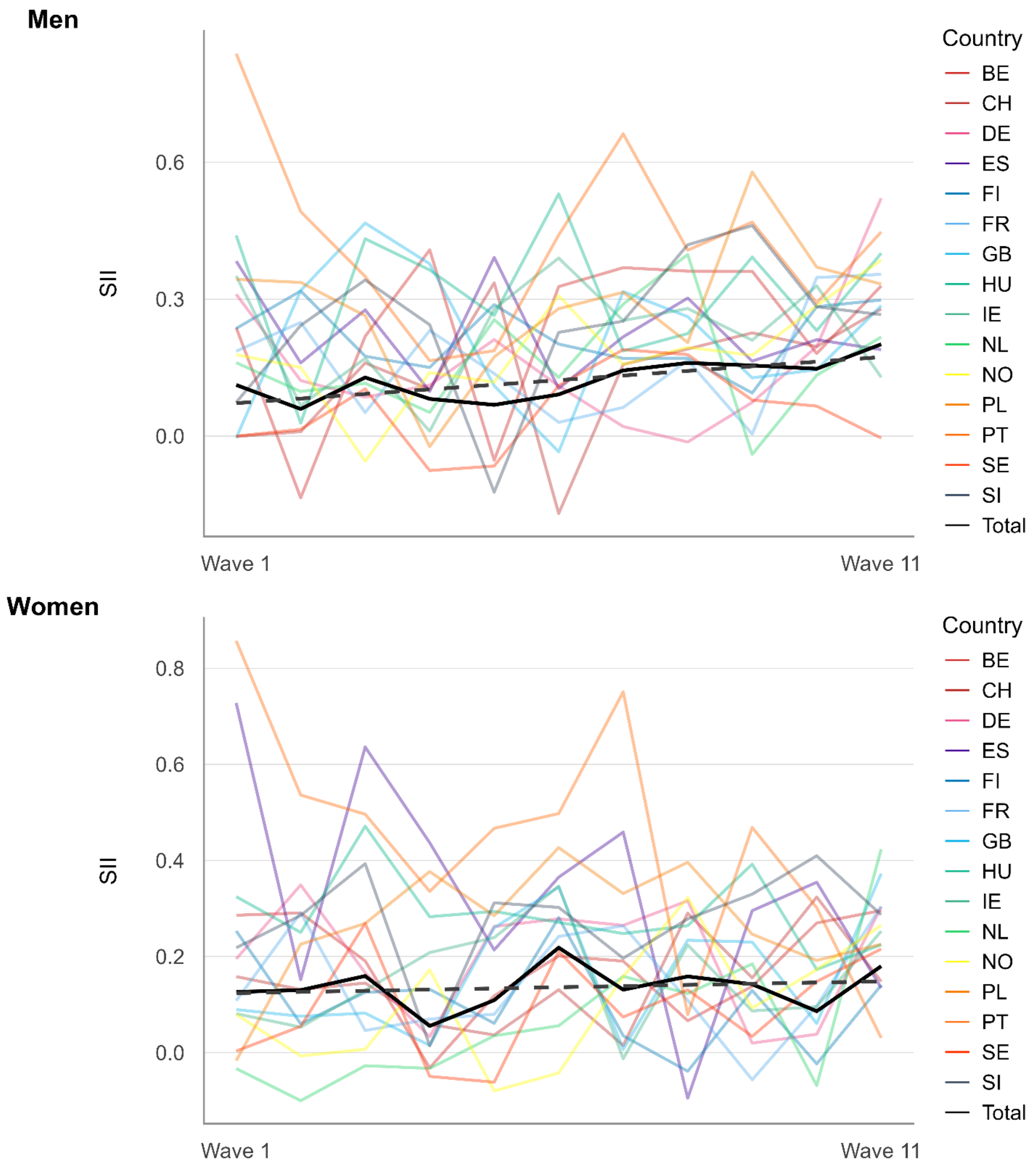

Figure S8: Relative Index of Inequality (RII) for disability by country and gender across all waves; age-standardized (European Social Survey, respondents aged 60+ years, waves 1 – 11, 2002 – 2023); higher RII values indicate greater inequality between education groups

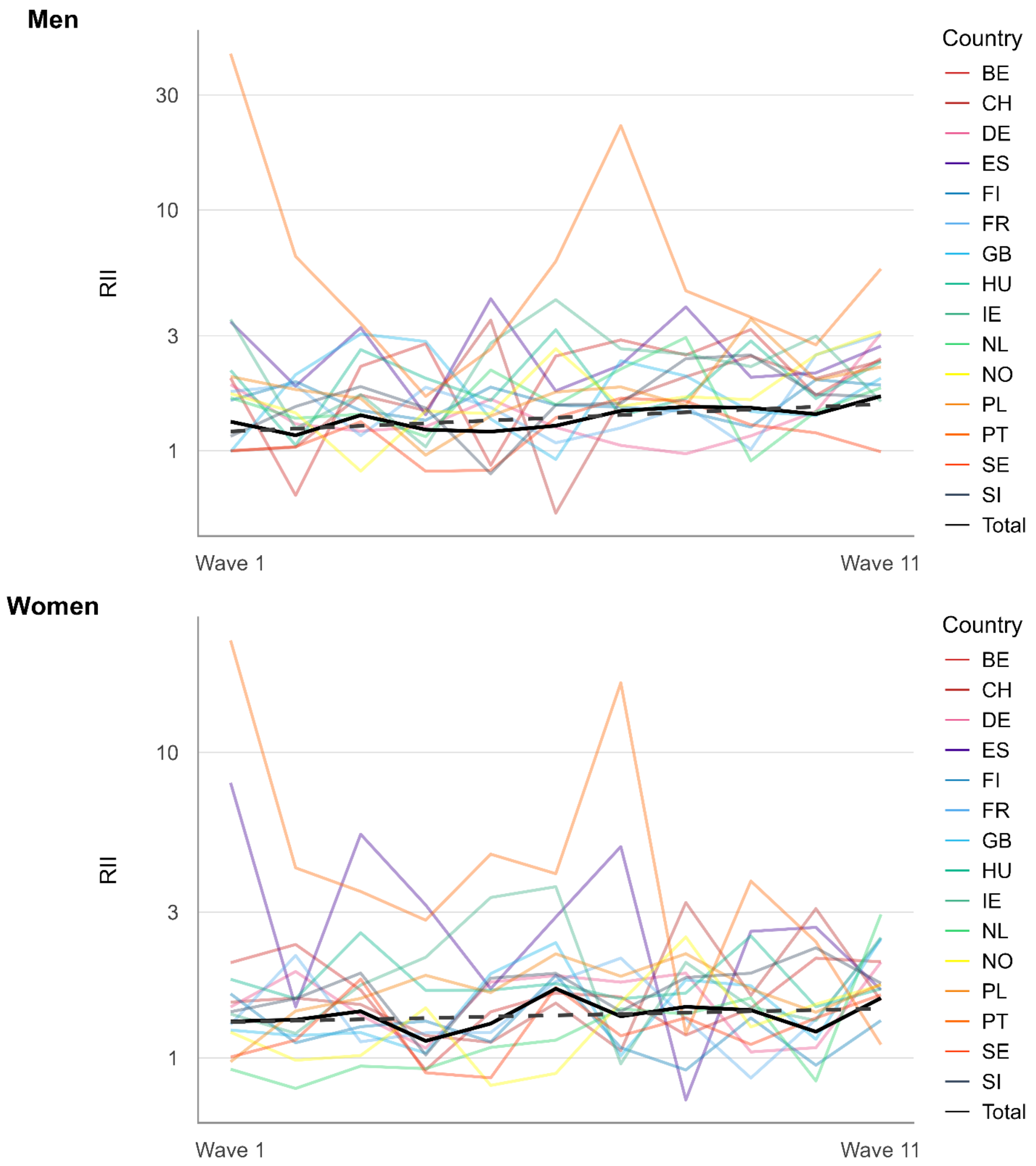

Supplement: Supplementary file 1 — Supplementary Information. [file 41598_2026_62524_MOESM1_ESM.pdf]
